# Effectiveness and safety of a shortened oral regimen for rifampicin- or multidrug-resistant TB

**DOI:** 10.5588/ijtldopen.25.0553

**Published:** 2026-03-13

**Authors:** E. Herrera-Flores, E. Shen, D. Vargas-Vasquez, F. Llanos-Tejada, Z. Ruiz-Vargas, J. Cornejo-García, D. Vela-Trejo, Z.M. Puyen-Guerra, M.C. Rojas, D.M. Guerra, M.L. Romo, J. Jimenez, E. Osso, L. Trevisi, A. LaHood, M.L. Rich, K.J. Seung, C.D. Mitnick, M.F. Franke, L. Lecca, V. Alarcon-Guizado

**Affiliations:** 1 Hospital Nacional Arzobispo Loayza, Lima, Peru;; 2 Harvard Medical School, Boston, MA, USA;; 3 Hospital Nacional Hipolito Unanue, Lima, Peru;; 4 Hospital Nacional Dos de Mayo, Lima, Peru;; 5 Hospital Nacional Maria Auxiliadora, Lima, Peru;; 6 Dirección de Prevención y Control de la Tuberculosis, Lima, Peru;; 7 Instituto Nacional de Salud, Lima, Peru;; 8 Socios en Salud, Lima, Peru;; 9 Department of Global Health and Social Medicine, Harvard Medical School, Boston, MA, USA;; 10 Division of Global Health Equity, Department of Medicine, Brigham and Women’s Hospital, Boston, MA, USA;; 11 Partners in Health, Boston, MA, USA;; 12 Department of Epidemiology, Harvard T.H. Chan School of Public Health, Boston, MA, USA.

**Keywords:** tuberculosis, Peru, quality of life, dyspnoea, culture conversion, adverse events, extensive TB disease

## Abstract

**BACKGROUND:**

Multidrug-resistant or rifampicin-resistant TB (MDR/RR-TB) poses significant challenges to patients, providers, and programmes. We evaluated a 9-month, 5-drug all-oral regimen implemented under operational conditions in Peru.

**METHODS:**

Between February and September 2023, we enrolled 50 adults with confirmed pulmonary MDR/RR-TB in a prospective observational study conducted within Peru’s National Tuberculosis Programme. The regimen consisted of bedaquiline, linezolid, levofloxacin, clofazimine, and delamanid, administered for 9 months and potentially extended to 12 months. We describe the frequency of clinically relevant adverse events of special interest, sputum culture conversion, end-of-treatment outcomes, and changes in dyspnoea and quality of life.

**RESULTS:**

Of 50 participants, 24 (48%) were women, and median age was 28.5 years (interquartile range [IQR]: 23–59 years); 38 (76%) had cavitary disease, and 29 (58%) had bilateral disease. Adverse events were infrequent and manageable; only one case of linezolid-associated myelosuppression led to permanent drug discontinuation. Of 33 participants with positive sputum culture, 100% experienced culture conversion (median: 39 days, IQR: 31–61). Favourable end-of-treatment outcomes were observed in 40 (85.1%) (95% confidence interval: 72.3%–92.6%). Quality-of-life and dyspnoea scores improved significantly in those with treatment success.

**CONCLUSION:**

This 9-month oral regimen was effective and safe and improved patient-reported outcomes. These results support broader adoption in national TB programmes across Latin America and beyond.

Drug-resistant TB (DR-TB) represents a significant global public health challenge. Approximately 500,000 people fall ill from multidrug-resistant or rifampicin-resistant (MDR/RR) TB annually. As of 2021, the global treatment success rate across regimens was 68%.^1^ In Peru, which represents a quarter of all MDR/RR-TB cases in the Americas, the treatment of MDR/RR-TB represents a major burden on the health system, with high patient costs, high rates of loss to follow-up, and suboptimal treatment success rates.^2^ Several trials and observational studies on shortened 6- and 9-month oral regimens have reported similar or superior therapeutic efficacy compared to conventional 18- to 20-month regimens, with success rates consistently surpassing 80% and even 90% in some studies.^3-8^ These results led the WHO to recommend the use of new, shortened all-oral regimens over longer treatments. The 2025 guidelines endorsed several 6- to 9-month all-oral regimens for the treatment of MDR/RR-TB, renewing the call for operational research to provide supplementary evidence for these approaches.^9,10^ The scale-up of all-oral shortened treatment regimens across national TB programmes (NTPs) is a key strategy to improve clinical outcomes for MDR/RR-TB globally.^11^ To date, most evidence has been generated in Europe and South Africa, with a scarcity of data from high-burden regions of Latin America.

Here, we provide evidence on the safety and effectiveness of a 9-month oral regimen of bedaquiline (Bdq), linezolid (Lzd), levofloxacin (Lfx), clofazimine (Cfz), and delamanid (Dlm) for patients with fluoroquinolone-susceptible MDR/RR-TB, under operational research conditions.

## METHODS

We conducted a prospective observational study within the Peru National Tuberculosis Programme (NTP). We consecutively enrolled 50 adults (aged > 18 years) with confirmed pulmonary MDR/RR-TB from clinics at four national public sector referral hospitals between February 1 and September 30, 2023. We excluded individuals with contraindications, allergies, or resistance to regimen medications, and those with conditions that, per the consulting physician, could interfere with adherence or follow-up (for additional details, see Supplementary Data).

### Standardised all-oral 9–12-month treatment regimen

The Peru NTP determined the composition of the all-oral shortened MDR/RR-TB treatment based on prevailing drug resistance profiles and the intensity of use of anti-TB drugs over the past decade. This regimen aligned with the drug priority ranking defined by the WHO and included three Group A drugs (Bdq, Lzd, and Lfx), one Group B drug (Cfz), and one Group C drug (Dlm).^10^ The intended duration was nine months (40 weeks, 240 doses), but could be extended to 12 months (50 weeks, 300 doses) based on clinical discretion in patients with (a) persistence of cavitary disease, smear positivity or culture positivity after the third month of treatment, (b) radiographic evidence of extensive pulmonary disease with involvement of at least two lung fields, or (c) immunosuppressive comorbidities such as diabetes mellitus or HIV.

### Study procedures

Clinical monitoring was carried out as part of routine care, following standards of the Peru NTP.^12^ Screening for adverse events took place monthly and included a complete blood count, hepatic function, ECG, and clinical evaluation for colourblindness and peripheral neuropathy (for additional details, see Supplementary Data).

### Adverse events reporting

Adverse events of special interest (AESIs) were established based on the known toxicity profiles of medications used in the all-oral regimen, and included QTc interval prolongation, hepatotoxicity, myelosuppression, visual disturbance, peripheral neuropathy, and seizures. Severity grading was conducted based on pertinent criteria from the MSF Severity Grading Scale, and pre-defined thresholds (Supplementary Table S1) were used to determine clinical relevance.^13^

### Symptom and quality of life reporting

Dyspnoea was assessed at baseline and end-of-treatment using the modified Medical Research Council Dyspnoea Scale (mMRC).^14^ Participants self-reported quality of life using the EuroQol-5D (EQ-5D)^15^ at baseline, 4 months, and end-of-treatment. Participants’ subjective evaluation of their overall health state was collected at the same intervals using the EuroQol Visual Analogue Scale (EQ-VAS), a single numerical self-rating from 0 (worst possible health) to 100 (best possible health).

### Statistical analysis

Safety analyses were conducted among all participants; effectiveness analyses excluded individuals with baseline fluoroquinolone drug resistance detected after enrolment. Safety analyses described clinically relevant AESIs, including the percentage of participants experiencing ≥1 episode, median time to first occurrence, and incidence rate. For each pre-specified AESI, person-months at risk were calculated from start of treatment until the first adverse event of clinical relevance or the end of treatment, whichever occurred first. For those withdrawn from the study due to late identification of baseline fluoroquinolone resistance, person-time was counted from start of treatment until the date of exclusion. Effectiveness outcomes included time to sputum culture conversion,^16^ end-of-treatment outcomes, and change in patient-reported dyspnoea, quality of life, and rating of overall health state. End-of-treatment outcomes were determined by clinicians, in accordance with national treatment guidelines, and based on the 2020 WHO updated definitions for MDR/RR-TB treatment outcomes^17^ (for additional details, see Supplementary Data). Dyspnoea scores at the end of treatment were compared against those from baseline by the Wilcoxon signed-rank test. The percentages of participants reporting quality of life difficulties were compared at three time points (baseline, 4 months, and end-of-treatment) via McNemar tests of change. Self-reported health ratings were compared for interval changes (baseline to 4 months, 4 months to end-of-treatment, and baseline to end-of-treatment) using the Wilcoxon signed-rank. Primary analyses were conducted in STATA 18 (StataCorp LLC, College Station, TX) and validated by a second analyst.

### Ethical statement

This study was approved by the Committee of Ethics in Biomedical Investigation of the Peru National Institutes of Health and the Harvard Longwood Campus Institutional Review Board. All participants gave written informed consent.

## RESULTS

Of 50 participants, 24 (48%) were women, and median age was 28.5 years (Table 1). At baseline, 29 (58%) had bilateral pulmonary disease, 38 (76%) had cavitary disease, and 9 (18%) had a body mass index less than 18.5 kg/m^2^. Concomitant cavitary disease and a sputum smear grade of at least 2+ was present in 15 (31%) patients. Bilateral cavitary disease (i.e., extensive disease as defined by the WHO) was observed in 25 (50%) patients.

**Table 1.** Baseline characteristics of individuals who initiated a shortened all-oral regimen for MDR/RR-TB in Peru (N = 50).

Characteristics	n (%)
Age, median (IQR; range)	28.5 (23–46; 18–77)
Female	24 (48.0)
Married or living together	19 (38.0)
Unemployed	40 (80.0)
Self-reported alcohol use	5 (10.0)
Self-reported cigarette use (N = 49)	2 (4.1)
Living with HIV	4 (8.0)
Diabetes	6 (12.0)
Hepatitis C	0 (0.0)
Low BMI (<18.5 kg/m^2^)	9 (18.0)
Bilateral disease	29 (58.0)
Cavitary disease	38 (76.0)
Fibrosis	30 (60.0)
Smear (N = 49)	
Negative	23 (46.9)
Scanty	1 (2.0)
1+	8 (16.3)
2+	11 (22.4)
3+	6 (12.2)
Culture positive (N = 48)	36 (75.0)
Baseline cavitary disease and smear grade > 2+ (N = 49)	15 (30.6)
Baseline cavitary disease and bilateral lung disease	25 (50.0)
Extra-pulmonary disease	1 (2.0)
Previous treatment with first-line drugs	9 (18.0)

MDR/RR-TB = multidrug-resistant or rifampicin-resistant TB; IQR = interquartile range, shown as 25th percentile, 75th percentile; BMI = body mass index.

### Safety

Adverse event monitoring occurred regularly at monthly intervals (Supplemental Data Table S2). Clinically relevant AESIs were infrequent (Table 2), and only one clinically relevant AESI led to subsequent permanent drug discontinuation. Peripheral neuropathy was experienced by three participants (6%), with one participant experiencing a grade 2 event and two participants experiencing grade 3 events. No permanent drug discontinuation occurred in these cases. Grade 3 myelosuppression was experienced by one participant (2%), resulting in the subsequent discontinuation of linezolid. Clinically relevant QTc prolongation, hepatotoxicity, optic neuritis, and seizures were not observed. Gastrointestinal intolerance, though not an AESI, resulted in the discontinuation of levofloxacin in six participants (12%). Of the six participants in whom levofloxacin was discontinued, four were subsequently switched to moxifloxacin with successful tolerance of this agent until the end of treatment. One participant completed treatment on the remaining four drugs without a fluoroquinolone. One participant had gastrointestinal intolerance to both levofloxacin and moxifloxacin and was switched to a longer individualised regimen.

**Table 2.** Frequency, months to first occurrence, and incidence rate of clinically relevant adverse events of special interest, among individuals who initiated a shortened all-oral regimen for MDR/RR-TB in Peru (N = 50).

Clinically relevant AESI	Frequency of >1 occurrence of clinically relevant AESI, n (%)	Months to first occurrence of clinically relevant AESI, median (range)	Rate of clinically relevant AESI/1,000 person-months (95% CI)	Events resulting in medication permanent discontinuation, n
Hepatotoxicity	0 (0.0)	—	—	—
Myelosuppression	1 (2.0)	0.5	2.4 (0.0–12.9)	1
Optic neuritis	0 (0.0)	—	—	—
Peripheral neuropathy	3 (6.0)	2.5 (2.3–2.6)	7.1 (0.0–20.4)	0
QTc prolongation	0 (0.0)	—	—	—
Seizures	0 (0.0)	—	—	—

MDR/RR-TB = multidrug-resistant or rifampicin-resistant TB; AESI = adverse event of special interest; IQR = interquartile range, represented as 25th percentile, 75th percentile; CI = confidence interval. Confidence intervals calculated using Fisher's exact test.

### Effectiveness

**Figure 1.**
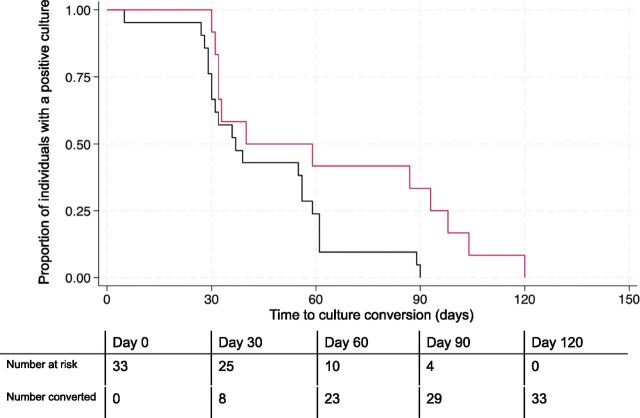
Kaplan-Meier curve of time to culture conversion among individuals who initiated a shortened all-oral regimen for multidrug-resistant or rifampicin-resistant TB with a positive baseline sputum culture, stratified by baseline cavitary disease with smear grade of >2+ (N = 33). Red line represents participants with both baseline cavitary disease and smear grade of 2+ or 3+ (n = 12); Black line represents participants who do not have both baseline cavitary disease and smear grade of 2+ or 3+ (n = 21).

Three of the 50 participants (6%) were excluded from effectiveness analyses due to detection of baseline fluoroquinolone resistance after enrolment (at days 14, 28, and 40) (Supplemental Data Figure S1). Of the remaining 47 participants, 45 (96%) had a baseline culture result, and this was positive in 33 (73%). Of participants with a positive baseline culture, all achieved culture conversion. The median time-to-conversion was 39 days (25th–75th percentile: 31–61 days), with a maximum time of 120 days (Supplementary Data Figure S2). Participants with baseline concomitant cavitary disease and smear grade of 2+ or 3+ (n = 12, 36%) experienced a longer time to culture conversion (log-rank *P* = 0.036), with a median of 49.5 days (25th–75th percentile: 32–95.5 days), as compared to 37 days (25th–75th percentile: 30–59 days) among those who did not present both of these disease characteristics (Figure 1). Favourable outcomes were observed in 40 of 47 participants (85.1%, 95% confidence interval [CI]: 72.3%–92.6%) (Table 3), of whom 39 were documented cured. Among unfavourable outcomes, there were two deaths (4.3%) and three losses to follow-up (6.4%). Treatment failed in one patient and was extended beyond 12 months in a second participant due to fluoroquinolone suspension from gastrointestinal intolerance. Of the two deaths, one was a homicide and one due to distributive shock and acute respiratory failure from advanced HIV and TB. The median treatment duration was 9.2 months (25th–75th percentile: 9.1–10.3 months).

**Table 3.** End-of-treatment outcomes among individuals who initiated a shortened all-oral regimen for MDR/RR-TB in Peru (N = 47).

Treatment outcomes	N (%) (95% CI)
Favourable	40 (85.1) (72.3–92.6)
Cured	39 (83.0)
Completed	1 (2.1)
Unfavourable	7 (14.9) (7.4–27.7)
Died	2 (4.3)
Treatment failure	1 (2.1)
Treatment extended > 12 months	1 (2.1)
Lost to follow-up	3 (6.4)

MDR/RR-TB = multidrug-resistant or rifampicin-resistant TB; CI = confidence interval.

### Dyspnoea

Self-reported dyspnoea was generally mild at baseline, with the majority (n = 49, 98%) of participants describing symptoms from grade 0 to grade 2. Among those with favourable end-of-treatment outcomes, mean change in dyspnoea score from baseline to end-of-treatment was −0.38 (*P* = 0.0002; Supplementary Data Table S3).

### Quality of life

**Figure 2.**
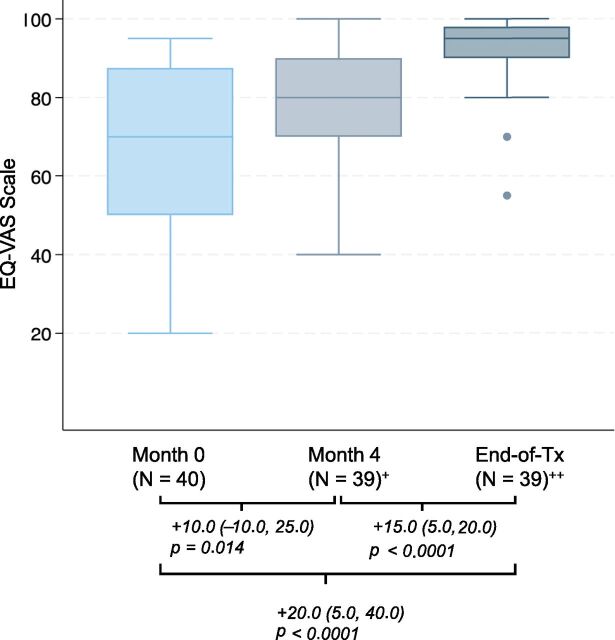
Boxplot of self-reported overall health scores among individuals who initiated a shortened all-oral regimen for multidrug-resistant or rifampicin-resistant TB with a favourable end-of-treatment outcome, STEM-TB Peru. Values under brackets are formatted as: + median change in score (25th percentile, 75th percentile). Wilcoxon signed-rank *P* value. ^+^One individual with favourable outcome status did not have a documented 4-month quality-of-life assessment; ^++^one individual with favourable outcome status did not have a documented end-of-treatment quality-of-life assessment.

Participants were most likely to report concerns in pain/discomfort and anxiety/depression domains (n = 32, 64%, and n = 30, 60%, reported any baseline symptoms, respectively). Participant functionality on dimensions of mobility (n = 8, 16%, reported any symptoms), daily activities (n = 9, 18%, reported any limitations), and self-care (n = 1, 2%, reported any limitations) was high at intake. Among those with favourable end-of-treatment outcomes, there was a decrease in reported pain or discomfort, with only 9 (23%) reporting any pain or discomfort by end-of-treatment (McNemar’s *P* = 0.002) (Supplementary Data Table S4). A reduction was also observed for anxiety and depression, which was reported in 16 (41%) by end-of-treatment (McNemar’s *P* = 0.083). Those with a favourable end-of-treatment outcome also reported improvement in self-rated overall health scores, with significant increases from baseline to 4 months of treatment (median, +10.0; Wilcoxon *P* = 0.014), and from 4 months until end-of-treatment (median, +15.0, Wilcoxon *P* < 0.0001) (Figure 2, Supplemental Data Table S5).

## DISCUSSION

Patients who received a 9-month, oral regimen comprising bedaquiline, linezolid, levofloxacin, clofazimine, and delamanid in Peru demonstrated rates of treatment success comparable to those from other shortened regimens containing bedaquiline, linezolid, and a fluoroquinolone. This includes the BEAT-TB trial, which recently reported a treatment success rate of 86% for a treatment strategy consisting of the same five medications used for 6 months. Importantly, the BEAT-TB trial used an adaptive design whereby levofloxacin or clofazimine was suspended based on fluoroquinolone susceptibility results.^7^ While the BEAT-TB strategy demonstrated non-inferiority to the South African standard overall, the risk difference for an unfavourable end-of-treatment outcome was qualitatively different between participants with fluoroquinolone susceptibility (−4.9%, 95% CI: −13.3% to 3.5%) and those with fluoroquinolone resistance (7.6%, 95% CI: −10.2% to 25.3%), raising questions about the optimal treatment strategy for the universe of patients seen in a TB programme.^7^

A potential benefit of five-drug regimens is that a drug may be removed, whether due to adverse reactions or fluoroquinolone resistance, without significantly compromising overall regimen efficacy. This contrasts with the three- and four-drug BPaL/M regimens, in which discontinuing linezolid reduces the regimen to two or three agents, potentially diminishing efficacy and requiring a switch to a longer, individualised regimen.^3,6,18^ In 2025, the WHO began recommending the BEAT-TB strategy for patients with MDR/RR-TB in whom fluoroquinolone resistance is unknown. That is, patients initiate the five-drug regimen, with subsequent tailoring based on whether the TB isolate is resistant (in which case levofloxacin may be discontinued) or susceptible (in which case clofazimine may be discontinued).^19^ However, given the pragmatic limitations of conducting this additional resistance testing in many high-burden settings with limited diagnostic infrastructure, the appropriate regimen for patients without fluoroquinolone susceptibility testing has not been well established under this framework. Our results imply that, even in cases when fluoroquinolone resistance testing cannot be completed, the full five-drug regimen is generally well tolerated for 9 months and may thus be a suitable option for patients with limited clinical data regarding underlying resistance.

The incidence of clinically significant adverse events was low and generally consistent with what has been reported with other bedaquiline-based shortened regimens.^3‐6^ Linezolid-related toxicity was notably infrequent. Although underreporting of linezolid toxicity is possible, compliance with routine monitoring procedures, including serial clinical evaluations and laboratory tests, was high. The widespread use of WHO-approved rapid molecular diagnostics has contributed to opportune diagnosis and treatment of MDR/RR-TB, resulting in fewer patients with advanced, undernourished, or systemically fragile conditions that may predispose to linezolid toxicity. Apart from the known toxicities associated with chronic exposure to linezolid, the medications used in this regimen have excellent tolerability profiles in the context of shortened treatment durations.^4,8,18^ AESIs did not appear to compromise the continuity or effectiveness of the regimen for the vast majority of patients; only one individual discontinued medication (due to linezolid-related myelosuppression). Notably, no cases of clinically relevant QTc prolongation or liver toxicity were reported, although this may be due to the small size of the cohort. The low frequency of QTc prolongation > 500 ms aligns with the preponderance of data on all-oral shortened regimens. Finally, although not a pre-defined AESI, gastrointestinal intolerance associated with levofloxacin did result in several drug suspensions or discontinuations. These events reassuringly did not compromise final treatment outcomes for the majority of affected individuals, with only one of six participants subsequently switching to a longer regimen.

This study was implemented within the Peru NTP and under real-world conditions in Peru’s public health system. This design enabled the establishment of implementation pathways for shortened oral regimens within the broader context of the Peru NTP. The low frequency of loss to follow-up aligns with the growing body of evidence suggesting that shortened oral regimens facilitate treatment completion and may thus offer a secondary benefit of interrupting ongoing transmission of drug-resistant strains of *Mycobacterium tuberculosis.*^20^ TB is known to adversely impact quality of life.^21^ In our cohort, patients who were successfully treated experienced improved health-related quality of life, particularly along the dimensions of pain/discomfort and anxiety/depression. Further improvement of recovery along the dimension of anxiety/depression may require addressing the effects of post-TB sequelae, or psychological and socio-economic factors affected by TB disease, and should be examined in future studies of post-TB lung disease.^22–24^

Our results have several limitations, including the small cohort size, exclusion of patients with prior exposure to second-line drugs, and exclusion of a small number (unenumerated) of individuals who may have faced significant challenges adhering to treatment. Further study is warranted of strategies to optimise treatment for fluoroquinolone-resistant TB. Additional areas for future research include examining whether other drug combinations or extension of 6-month regimens to 9 months in patients with advanced disease or comorbidities that may affect the sterilisation of pulmonary TB lesions and improve outcomes, particularly by reducing relapse rates and the risk of resistance amplification. Also important is a fuller understanding of the factors that cause linezolid toxicity and may lead to heterogeneity across populations.

## CONCLUSIONS

Under operational research conditions within Peru’s NTP, a 9–12-month all-oral 5-drug regimen demonstrated high treatment success, good tolerability, and improved patient-reported outcomes. Effectiveness and safety were comparable to other WHO-endorsed shortened regimens. This regimen offers a valuable alternative for patients unable to receive pretomanid – such as children, pregnant individuals, or those with intolerance – and may also provide greater flexibility in cases of advanced disease, comorbidities, or fluoroquinolone resistance. Given its favourable safety profile and robustness against treatment interruptions, further research should explore whether this regimen can reduce relapse and resistance amplification in high-risk populations. These results support the broader integration of this five-drug regimen into NTP options, particularly in settings where pretomanid access remains limited.

## Supplementary Material




