# Supplementary material for: Effectiveness and safety of a shortened oral regimen for rifampicin- or multidrug-resistant TB
Source: IJTLD Open. 2026 Mar 13;3(3):144–50. doi: 10.5588/ijtldopen.25.0553 (PMC12991560; doi:10.5588/ijtldopen.25.0553)
Supplement: Supplementary file 1 [file ijtldopen25-0553_supplementarydata1.pdf]

Supplemental Material

Effectiveness and Safety of a Shortened Oral Regimen for Rifampicin- or Multidrug-Resistant Tuberculosis in Peru

1. Edwin Herrera-Flores, MD, Hospital Nacional Arzobispo Loayza. Lima, Peru.
2. Edmund Shen, BA, Harvard Medical School. Boston, Massachusetts, USA.
3. Dante Vargas-Vasquez, MD, Hospital Nacional Hipolito Unanue. Lima, Peru.
4. Félix Llanos-Tejada, MD, Hospital Nacional Dos de Mayo. Lima, Peru.
5. Zully Ruiz-Vargas, MD, Hospital Nacional Maria Auxiliadora. Lima, Peru.
6. José Cornejo-García, MD, Hospital Nacional Arzobispo Loayza. Lima, Peru.
7. Dennise Vela-Trejo, PN, Dirección de Prevención y Control de la Tuberculosis. Lima, Peru.
8. Zully M. Puyen-Guerra, PhD, Instituto Nacional de Salud. Lima, Peru.
9. Maricela Curisinche Rojas, PhD, Instituto Nacional de Salud. Lima, Peru.
10. Dalia M. Guerra, RN, Socios en Salud. Lima, Peru.
11. Matthew L. Romo, PhD, Department of Global Health and Social Medicine, Harvard Medical School. Boston, Massachusetts, USA.
12. Judith Jimenez, MPH, Socios en Salud. Lima, Peru.
13. Elna Osso, RN, MPH, Department of Global Health and Social Medicine, Harvard Medical School. Boston, Massachusetts, USA.
14. Letizia Trevisi, PhD, Department of Global Health and Social Medicine, Harvard Medical School. Boston, Massachusetts, USA.
15. Allison LaHood, MS, Department of Global Health and Social Medicine, Harvard Medical School. Boston, Massachusetts, USA.
16. Michael L. Rich, MD, MPH, Division of Global Health Equity, Department of Medicine, Brigham and Women's Hospital. Boston, Massachusetts, USA. Partners In Health, Boston, Massachusetts, USA.
17. Kwonjune Justin Seung, MD, Division of Global Health Equity, Department of Medicine, Brigham and Women's Hospital. Boston, Massachusetts, USA. Partners In Health, Boston, Massachusetts, USA.
18. Carole D. Mitnick, ScD, Department of Global Health and Social Medicine, Harvard Medical School. Boston, Massachusetts, USA. Partners In Health, Boston, Massachusetts, USA.
19. Molly F. Franke, ScD, Department of Global Health and Social Medicine, Harvard Medical School. Boston, Massachusetts, USA. Department of Epidemiology, Harvard T.H. Chan School of Public Health, Boston, Massachusetts, USA.
20. Leonid Lecca, MD, MSc, Socios en Salud. Lima, Peru. Department of Global Health and Social Medicine, Harvard Medical School. Boston, Massachusetts, USA.
21. Valentina Alarcon-Guizado, MD, MPH, Dirección de Prevención y Control de la Tuberculosis. Lima, Peru.

Supplemental Table 1. Adverse Events of Special Interest and Severity Scale for Clinically Relevant Adverse Events of Special Interest, STEM-TB Peru

AESI	Severity Scale or "Other" Terms Included	Grade(s)	Definition for Minimum Grade to Be Considered Clinically Relevant
Hepatotoxicity	Alanine aminotransferase (ALT or SGPT) increased	≥ 3	Grade 3: alanine aminotransferase and/or aspartate aminotransferase >5 times the upper limit of normal
	Aspartate aminotransferase (AST or SGOT) increased	..	..
	Hepatotoxicity	..	..
	Hepatitis	..	..
Myelosuppression	Anemia	Anemia ≥ 3	Grade 3: hemoglobin < 7.9 g/dL
	Platelets decreased	Thrombocytopenia ≥ 3	Grade 3: platelets decreased <50,000/mm^3
	White blood cells decreased	Leukopenia ≥ 3	Grade 3: white blood cells decreased <2000/mm^3
	Lymphocyte count decreased	Lymphocytopenia ≥ 3	Grade 3: lymphocytes decreased <500/mm^3
	Absolute neutrophil count	Neutropenia ≥ 2	Grade 2: absolute neutrophile count <750/mm^3
	Pancytopenia	Pancytopenia ≥ 2	Grade 2: any combination of the above
Optic neuritis	Optic nerve disorder (optic neuritis)	All grades	Grade 1: clinical diagnosis, no symptoms
Peripheral neuropathy	Neurosensory disorders	All grades	Grade 1: mild impairment or discomfort / Brief Peripheral Neuropathy Screen subjective sensory neuropathy score 1-3 on any side
	Paresthesia (burning, tingling, etc.)	..	..
QT prolongation	Electrocardiogram QT-corrected interval prolonged	≥ 3	Grade 3: QTc≥501 msec, no symptoms
Seizures	Seizure	All grades	Grade 1: brief partial seizure, no loss of consciousness

Abbreviations: AESI, adverse event of special interest; QTc, QT interval corrected by the Fridericia method; SGOT, serum glutamic oxaloacetic transaminase; SGPT, serum glutamate pyruvate transaminase; ALT, alanine aminotransferase; AST, aspartate aminotransferase

Supplemental Table 2. Frequency of Baseline and On-Treatment Monitoring Evaluations for Individuals Who Initiated a Shortened All-Oral Regimen for MDR/RR-TB, STEM-TB Peru (N = 50)

Evaluation	median (IQR)
Hemoglobin	11 (11, 13)
Hepatic function testing – ALT	11 (11, 12)
Hepatic function testing – AST	11 (11, 12)
ECG	12 (12, 13)
Visual acuity screening	10 (9, 11)
Colorblindness screening	10 (9, 11)
Peripheral neuropathy screening	10 (9, 11)

IQR: interquartile range, shown as 25th percentile, 75th percentile.
Includes all evaluations obtained before the end-of-treatment date.

Supplemental Table 3. Self-Reported Dyspnea Scale Scores, at Baseline and End-of-Treatment Among Individuals Who Initiated a Shortened All-Oral Regimen for MDR/RR-TB with a Favorable End-of-Treatment Outcome, STEM-TB Peru (N = 39)

	Median	Mean	Standard Deviation	Minimum	Maximum	IQR
Baseline (n=39)	0	0.56	0.64	0	2	0 – 1
End-of-treatment (n=39)	0	0.18	0.51	0	2	0 – 0

IQR: interquartile range, shown as 25th percentile, 75th percentile

Dyspnea scores were reported on range of 0 (only breathless with strenuous exercise) to 4 (too breathless to leave the house, or breathless while dressing and undressing), per grading from the single-question mMRC dyspnea scale. Median change in dyspnea score from baseline to end-of-treatment was 0 (25th – 75th percentile: -1 to 0); Wilcoxon signed-rank p = 0.0005.

Supplemental Table 4. Self-Reported Quality of Life Score According to the 5 Dimensions of the EQ-5D Survey Measured at Baseline, 4 Months of Treatment, and End-of-Treatment Among Individuals Who Initiated a Shortened All-Oral Regimen for MDR/RR-TB with a Favorable End-of-Treatment Outcome, STEM-TB Peru

	**Pre-Treatment (N = 40)**	**Treatment Month 4 (N = 39)[+]**	**End-of-Treatment (N = 39)[++]**
**Mobility**			
No problems	35 (88)	32 (82)	33 (85)
Mild problems	5 (12)	6 (15)	6 (15)
Moderate problems	0	1 (3)	0
Severe problems	0	0	0
Unable to walk	0	0	0
**Daily Activities**			
No problems	36 (90)	35 (90)	32 (82)
Mild problems	4 (10)	4 (10)	7 (18)
Moderate problems	0	0	0
Severe problems	0	0	0
Unable to perform	0	0	0
**Self-care**			
No problems	39 (98)	37 (95)	38 (97)
Mild problems	1 (2)	2 (5)	1 (3)
Moderate problems	0	0	0
Severe problems	0	0	0
Unable to wash or dress	0	0	0
**Pain & Discomfort [+++]**			
No pain	16 (40)	28 (71)	30 (77)
Mild pain	17 (43)	8 (21)	6 (15)
Moderate pain	6 (15)	3 (7)	3 (8)
Severe pain	1 (2)	0	0
Extreme pain	0	0	0
**Anxiety & Depression ***			
None	17 (43)	24 (62)	23 (59)
Mild	20 (50)	14 (36)	14 (36)
Moderate	3 (7)	1 (2)	2 (5)
Severe	0	0	0
Extreme	0	0	0

[+] One individual with favorable outcome status did not have a documented 4-month quality-of-life assessment

[+++] McNemar tests of change in Pain / Discomfort response distribution for baseline vs. 4-months ($X^2 = 10.29$, $p = 0.0013$), and baseline vs. end-of-treatment ($X^2 = 9.80$, $p = 0.002$).

* McNemar tests of change in Anxiety / Depression response distribution for baseline vs. 4-months ($X^2 = 3.27$, $p = 0.071$), and baseline vs. end-of-treatment ($X^2 = 3.00$, $p = 0.083$).

Supplemental Table 5. Self-Reported Overall Health Rating According to the EQ-VAS Scale at Baseline, 4 months of Treatment, and End-of-Treatment Among Individuals Who Initiated a Shortened All-Oral Regimen for MDR/RR-TB with a Favorable End-of-Treatment Outcome, STEM-TB Peru

	Median	Mean	Standard Deviation	Minima	Maxima	IQR
Baseline (n=40)	70	68	19.8	20	95	50 – 87.5
4 Months (n=39)[+]	80	76.8	14.0	40	100	70 – 90
End-of-treatment (n=39)[++]	95	91.9	9.4	55	100	90 – 98

IQR: interquartile range, shown as 25th percentile – 75th percentile

Health ratings were self-reported on a range of 0 (worst imaginable health state) to 100 (best imaginable health state) according to the single-item EQ-VAS scale.
[+] One individual with favorable outcome status did not have a documented 4-month quality-of-life assessment.
[++] One individual with favorable outcome status did not have a documented end-of-treatment quality-of-life assessment

Supplemental Table 6. Standardized Drug Dosing for Individuals Who Initiated a Shortened All-Oral Regimen for MDR/RR-TB, STEM-TB Peru

Medication	Adult Body Weight: 30kg or more
Bedaquiline (100 mg tablets)	400 mg daily for 14 doses, followed by 200 mg three times per week
Delamanid (50 mg tablets)	100 mg two times per day
Linezolid (600 mg tablets)[†]	600 mg once per day*
Levofloxacin (250 mg or 500 mg tablets)	750 mg total daily
Clofazimine (100 mg gel capsules)**	100 mg daily

* Linezolid dosing may be reduced to 600 mg three times per week, or 300 mg daily, in patients who develop linezolid-associated peripheral neuropathy
** Clofazimine dosing may be initiated with 200 mg daily for nine weeks, and subsequently decreased to 100 mg daily for the remainder of treatment duration
[†] Participants may receive pyridoxine 50mg daily in association with linezolid administration

Supplemental Figure 1. Flowchart of Individuals Who Initiated a Shortened All-Oral Regimen for MDR/RR-TB, STEM-TB Peru

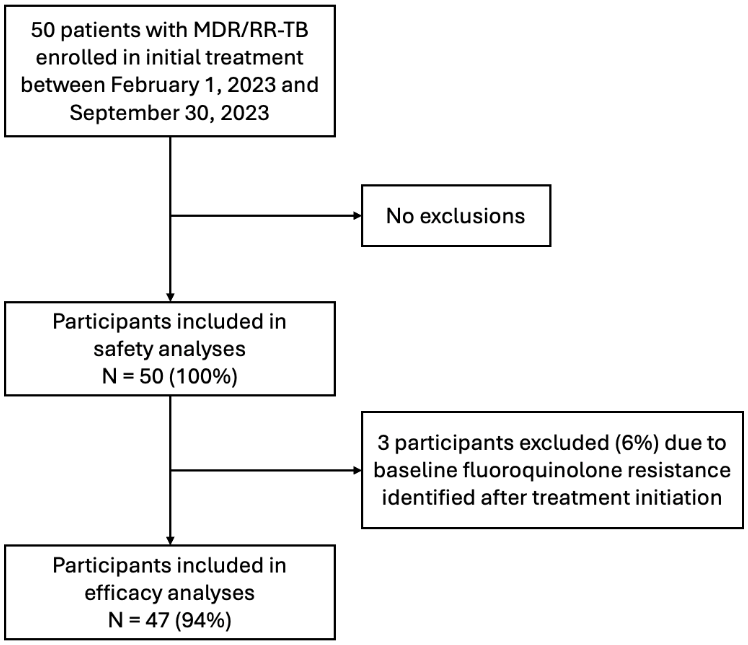

Supplemental Figure 2. Kaplan-Meier Curve of Time to Culture Conversion Among Individuals Who Initiated a Shortened All-Oral Regimen for MDR/RR-TB with a Positive Baseline Sputum Culture, STEM-TB Peru (N = 33)

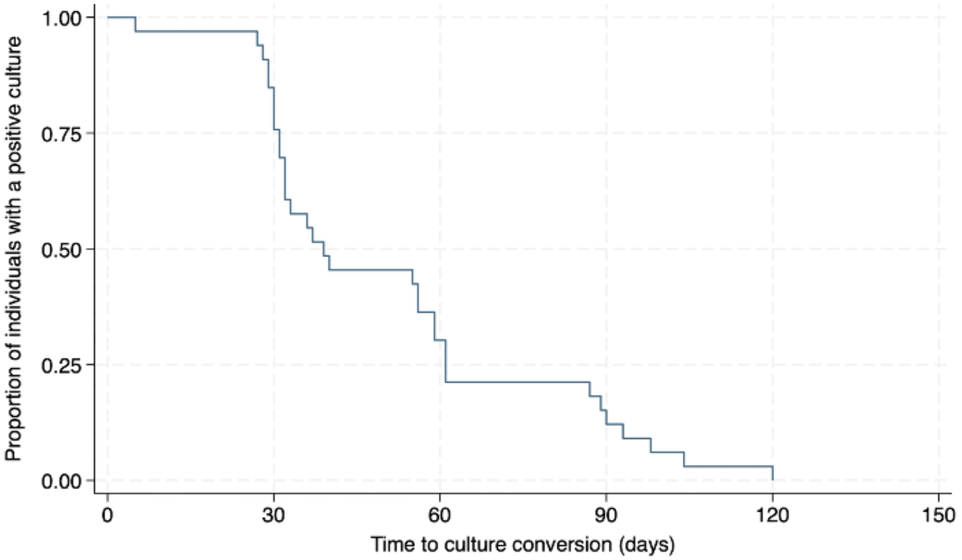

	Day 0	Day 30	Day 60	Day 90	Day 120
Number at risk	33	25	10	4	0
Number converted	0	8	23	29	33

Among the 36 individuals with positive baseline cultures, three individuals were subsequently withdrawn from the study due to initial fluoroquinolone resistance, and therefore excluded from effectiveness analyses. Among the remaining individuals (N = 33), 100% achieved culture conversion during treatment.

Supplementary Methods Information

Exclusion criteria

Patients were excluded if they met any of the following criteria: contraindication or known hypersensitivity to any drugs comprising the standardized regimen; a strain of *Mycobacterium tuberculosis* resistant to any drug in the regimen; previous MDR/RR-TB treatment with second-line drugs; known contact with someone with fluoroquinolone-resistant TB; a corrected QT interval (according to Fridericia's formula) ≥500 milliseconds, baseline AST or ALT levels >3 times the upper normal limit; a creatinine clearance <30 mL/min per 1·73 m^2 of body surface area; pregnancy; or any condition that, per the consulting physician, could interfere with follow-up or put the patient's safety at risk.

Definitions

Baseline cultures and smears were from samples collected up to 180 days before treatment initiation. Patients were considered 'cured' if treatment was completed with no evidence of failure and with the last three cultures negative. Patients were assigned an outcome of 'completed' if treatment was completed as recommended, with no evidence of failure, and in the absence of three consecutive negative cultures. Treatment failure was defined as termination of the regimen or a permanent change of at least two anti-TB drugs from the initial five-drug regimen due to lack of conversion after four months of therapy, bacteriological reversion, evidence of acquired resistance, or adverse drug reactions. Loss to follow-up was defined as a treatment interruption lasting 30 days or more. Individuals assigned an end-of-treatment status of "cured" or "completed" were considered to have a favorable end-of-treatment outcome. Sputum culture conversion among those with a positive baseline sputum culture was defined as two consecutive negative cultures, from samples collected at least seven days apart. Time to culture conversion was calculated as the time from treatment initiation until the sample collection date of the first of the two consecutive negative cultures.

Study Procedures

The administration and clinical monitoring of the treatment were carried out as part of routine care, following the standards of the Peru NTP. Screening for adverse events took place monthly during treatment and included a complete blood count, hepatic function (AST / ALT), ECG, and clinical evaluation for colorblindness (Ishihara Color Plates) and peripheral neuropathy (Brief Peripheral Neuropathy Screen).

Medications were dosed according to a standardized scheme and administered via directly (or video-) observed therapy six days per week, in accordance with NTP guidelines (Supplemental Table 6). Enrolled participants who were found to have resistance to fluoroquinolones or any other drug in the regimen, from a baseline sputum sample, were subsequently excluded from the study and treated with longer individualized regimens in accordance with Peru national treatment guidelines. These individuals were included in safety analyses for the duration of receipt of the all-oral shortened regimen.

The baseline clinical evaluation included a comprehensive medical history and physical examination, collection of sputum, blood, and urine samples, pregnancy testing, an ECG, a chest x-ray, and assessments of visual acuity, color vision, and peripheral neuropathy. Sputum samples were evaluated via smear microscopy, culture, and drug susceptibility testing. Cultures were conducted in mycobacterial growth indicator tubes (MGIT) for treatment initiation and on Ogawa or Löwenstein-Jensen agar during follow-up. Rifampicin resistance was assessed via Xpert MTB/RIF Ultra or Hain GenoType Line Probe Assay MTBDRplus; second-line phenotypic DST was conducted using MGIT. Blood samples were used for a complete blood count and to evaluate serum electrolytes, serum glucose, hepatic function, thyroid function, HIV, and hepatitis serologies. Two weeks following treatment initiation and monthly thereafter, follow-up assessments consisted of evaluating new or worsening signs and symptoms, sputum smear microscopy and culture, and a physical examination.

Repeat chest x-rays and pregnancy testing were also completed at follow-up during the 3rd, 6th, and 9th months of treatment. Patients also received, as needed, social services, nutrition counseling and support, reproductive health services, and mental health screening as part of the routine integrated services offered by the Peru NTP.

Symptom and Quality of Life Reporting

Dyspnea was assessed at baseline and at the end of treatment using the modified Medical Research Council Dyspnea Scale (mMRC), a single-question, self-reported score ranging from 0 (only breathless with strenuous exercise) to 4 (too breathless to leave the house, or breathless while dressing and undressing).[15] We also collected participant self-assessments of quality of life (QoL) using the EuroQol-5D (EQ-5D), a five-question survey that assesses mobility, daily activities, capacity for self-care, pain/discomfort, and depression/anxiety. For each dimension, participants self-reported their level of difficulty as one of five categories, ranging from 'none' to 'extreme'.[16] EQ-5D responses were gathered at baseline, four months, and the end of treatment. Participants' subjective evaluation of their overall health state was collected at the same intervals using the EuroQol Visual Analogue Scale (EQ-VAS), which consisted of a single numerical self-rating from 0 (worst possible imaginable health) to 100 (best possible imaginable health).

Data management

All baseline and follow-up clinical data were recorded in the endTB electronic data system administered by Socios en Salud, Peru.
